# Incidence trends of aplastic anaemia in Kazakhstan: a nationwide study (2014–2024)

**DOI:** 10.3389/fmed.2026.1856990

**Published:** 2026-06-22

**Authors:** Zhanna Sarbassova, Ulday Akhmetova, Balnur Iskakova, Nargiz Nassyrova, Lyazzat Kosherbayeva, Nurgul Alekenova

**Affiliations:** 1 Kazakh-Russian Medical University, Almaty, Kazakhstan; 2 Marat Ospanov West-Kazakhstan Medical University, Aktobe, Kazakhstan; 3 Asfendiyarov Kazakh National Medical University, Almaty, Kazakhstan; 4 Farabi University, Almaty, Kazakhstan

**Keywords:** aplastic anaemia, epidemiology, incidence, Kazakhstan, temporal trends

## Abstract

**Background:**

Aplastic anaemia (AA) is a rare hematologic disorder with substantial morbidity and mortality. Limited data are available on its epidemiology in Central Asia, including Kazakhstan.

**Objective:**

To analyze temporal trends and regional variation in the incidence of AA in Kazakhstan from 2014 to 2024.

**Methods:**

A retrospective population-based longitudinal study was conducted using national electronic medical records. Incident cases of AA were identified using ICD-10 code D61 and categorized as idiopathic/unspecified or secondary. Incidence rates per 100,000 population were calculated annually and analyzed by region and disease subtype. Descriptive statistics were used to summarize demographic and clinical characteristics.

**Results:**

A total of 1,209 incident cases were identified during the study period. The median age was 26 years, with a slight female predominance (52.3%). Most cases were classified as idiopathic or unspecified (88.9%). The national incidence of AA increased from 0.21 per 100,000 population in 2014 to 0.82 per 100,000 in 2024. This increase was primarily driven by idiopathic cases, while secondary AA remained relatively stable and uncommon. Regional variation was observed, with the highest incidence in major urban centers (up to 1.48 per 100,000 in 2024), whereas other regions ranged from 0.3 to 0.9 per 100,000.

**Conclusion:**

The incidence of AA in Kazakhstan has increased over the past decade, largely due to a rise in idiopathic cases. Geographic disparities suggest differences in healthcare access and diagnostic capacity. Strengthening surveillance systems and improving equitable access to specialized care are essential for better detection and management of AA.

## Introduction

Aplastic anaemia (AA) is a rare, life-threatening hematologic disorder characterized by failure of the bone marrow to produce adequate numbers of blood cells, resulting in pancytopenia and leading to complications such as fatigue, infections, and bleeding. The condition is most commonly driven by immune-mediated destruction of hematopoietic stem and progenitor cells, although other causes such as drug toxicity, viral infections, radiation exposure, and inherited bone marrow failure syndromes have also been implicated (1, 2). Untreated AA is associated with high mortality. Nevertheless, major advances in immunosuppressive therapy (IST), including antithymocyte globulin and cyclosporine, as well as allogeneic hematopoietic stem cell transplantation (HSCT), have substantially improved prognosis and survival outcomes for patients with AA (3).

The epidemiology of AA demonstrates substantial geographic variation. In Western countries, the annual incidence is approximately 2–3 cases per million population, as reported in Sweden (2.35 per million/year) and Spain (2.83 per million/year) (4, 5). In contrast, several Asian countries, including Thailand and Taiwan, have reported higher incidence rates of AA, reaching 4.6 and 5.67 cases per million population per year, respectively, with the highest rates observed among individuals aged over 70 years (6–8). Despite advances in treatment, AA continues to be associated with significant morbidity and mortality. A 12-year multi centre study from East Malaysia reported a 5-year overall survival rate of 76.1%, with poorer survival observed among patients aged over 60 years and those with severe disease (9). Similarly, a retrospective study from Costa Rica demonstrated higher mortality among patients with very severe AA and those aged over 65 years, while treatment with rabbit antithymocyte globulin and cyclosporine was associated with favorable clinical outcomes (10). In South Africa, most patients presented with severe disease; however, immunosuppressive therapy achieved an 80.9% response rate, highlighting its effectiveness even in resource-limited settings (11). In contrast, studies from South and Southeast Asia showed younger disease onset and male predominance (12, 13).

Socioeconomic, environmental and genetic factors appear to play an important role, with increased risk in rural areas, including exposures to agricultural chemicals such as pesticides and fertilizers (13, 14). In addition, genetic predisposition is increasingly recognized as a key contributor to disease development (15). Furthermore, mutations in telomere-related genes, including TERT and TERC, provide additional evidence supporting the role of inherited genetic susceptibility in disease progression (16). These findings indicate that both immune dysregulation and inherited genetic variation contribute to the heterogeneity of AA.

In Kazakhstan, recent studies have identified strong associations between AA susceptibility and specific HLA Class II alleles, including variants within DQA1 and DPB1, highlighting the importance of region-specific genetic profiling (17). Despite these advances, comprehensive epidemiological data on AA in Kazakhstan remain scarce, particularly regarding temporal trends, geographic distribution, and differences between idiopathic and secondary forms of the disease. Furthermore, a bibliometric analysis of 3,221 publications on aplastic anemia published between 1980 and 2022 demonstrated that research in this field is predominantly concentrated in the United States, China, Japan, and several European countries, while data from Central Asia, including Kazakhstan, remain limited (18). This highlights an important gap in the current literature and underscores the need for population-based epidemiological studies in the region. Therefore, this study aims to describe temporal and geographic patterns in the incidence of AA in Kazakhstan from 2014 to 2024 and to assess differences between idiopathic/unspecified and secondary cases, providing a foundation for improved understanding, prevention, and management of AA in this population.

## Methods

### Study design and objective

This population-based longitudinal study examined temporal trends in the incidence of AA in Kazakhstan from 2014 to 2024. The primary objectives were to describe national and regional incidence patterns over time and to compare trends between idiopathic/unspecified and secondary forms of AA, with particular attention to geographic variation.

### Data sources and study population

Patient-level data were obtained from national electronic medical records covering all administrative regions of Kazakhstan. Diagnoses were identified using the *International Classification of Diseases, Tenth Revision* (ICD-10) classification system developed by the World Health Organization (19). AA was defined using ICD-10 code D61 and its subcategories, which include aplastic anaemia and related bone marrow failure syndromes. For analytical purposes, diagnoses were grouped into two categories to reduce potential misclassification and improve interpretability of incidence estimates:

- Idiopathic/unspecified AA, including idiopathic aplastic anaemia (D61.3), other specified aplastic anaemia (D61.8), and aplastic anaemia, unspecified (D61.9). These categories represented cases without a clearly documented identifiable cause within the administrative database.
- Secondary AA, including constitutional aplastic anaemia (D61.0), drug-induced aplastic anaemia (D61.1), aplastic anaemia due to other external agents (D61.2), and related subcategories representing cases with identifiable acquired or inherited causes as classified within the ICD-10 system.

Other available variables included year of diagnosis, region of residence, diagnosis category, sex, date of birth, diagnosis date, and unique patient identifiers. Population data were reshaped into long format to allow linkage with case counts by region and year. Incidence rates were calculated only for region–year combinations with valid population denominators. Region names were standardized and transliterated into English for consistency. No population imputation was performed. To ensure comparability over time and account for administrative restructuring, regions were grouped into six geographic macro-regions: *North*: Akmola, Kostanay, North Kazakhstan, Pavlodar; *South*: Almaty region, Zhambyl, Kyzylorda, Turkestan, Zhetisu; *East*: East Kazakhstan, Abai; *West*: West Kazakhstan, Atyrau, Mangystau, Aktobe; *Central*: Karaganda, Ulytau; *Major cities*: Almaty city and Astana city.

### Case ascertainment and data cleaning

The raw dataset initially contained 5,240 records. After restricting the dataset to diagnoses recorded from 1 January 2014 onward, 4,366 records remained. Records with missing key variables were removed, resulting in 4,245 observations. Duplicate entries referring to the same individual were identified using unique patient identifiers and removed. To ensure that only incident cases were included, individuals with a recorded aplastic anaemia diagnosis in any prior year were excluded. For each patient, only the first recorded diagnosis within the study period (2014–2024) was retained. The final analytic dataset included 1,209 incident cases. Annual numbers of incident cases were aggregated by calendar year, region, and diagnosis group.

### Statistical analysis

Descriptive statistics were used to summarize annual numbers of incident cases and incidence rates by diagnosis group at national and regional levels. In addition, bivariate analyses were performed to compare demographic and regional characteristics between idiopathic/unspecified and secondary AA groups using Pearson’s chi-square test for categorical variables and the Wilcoxon rank-sum test for the continuous variable age due to its non-normal distribution.

Annual incidence rates were calculated as:


$$\text{Incidence}\;\mathrm{per}\;100\;000=\frac{\text{Number of}\;\mathrm{new}\;\text{cases in}\;a\;\text{given year}}{\text{Population in that year}}\times 100\;000$$

Trends in incidence were examined over time by presenting annual incidence rates at the national level and stratified by region and diagnosis group (idiopathic/unspecified vs. secondary AA). Incidence trends were visualized using line graphs to illustrate changes across calendar years. All data management, calculations, and visualizations were performed using R statistical software (version 4.4.1; 26).

## Results

### Sample characteristics

Descriptive characteristics of incident AA cases in Kazakhstan, 2014–2024, are summarized in Table 1. The median age of cases was 26.7 years (IQR: 12.3–50.9), while the mean age was 31.9 ± 22.8 years. Slightly more than half of the cases were female (632; 52.3%), while 577 (47.7%) were male. The majority of cases were classified as idiopathic or unspecified AA (1,074; 88.9%), with secondary AA accounting for 135 cases (11.1%). Geographically, the highest number of cases was reported in major cities (338; 28%). These findings indicate that AA affects a wide age range, with idiopathic cases predominating, and that the highest incidence is observed in major urban centers. Bivariate analyses showed no statistically significant differences between idiopathic/unspecified and secondary AA groups with respect to age distribution (*p* = 0.065), sex (*p* = 0.147), or geographic macro-region (*p* = 0.657). These findings indicate that AA affects a wide age range, with idiopathic cases predominating, and that the highest incidence is observed in major urban centers.

**Table 1:** Demographic and clinical characteristics of incident aplastic anaemia cases in Kazakhstan, 2014–2024 by diagnostic group (*n* = 1,209).

Variables	Idiopathic/unspecified AA *n* = 1,074	Secondary AA *n* = 135	*p*-value
Age, years			0.065
Median (IQR)	26.7 (12.3–50.9)		
Mean ± SD	31.9 ± 22.8		
Sex, *n* (%)			0.147
Female	553 (51.5)	79 (58.5)	
Male	521 (48.5)	56 (41.5)	
Geographic macro-regions, *n* (%)			0.657
Almaty and Astana cities*	188 (17.5)	20 (14.8)	
Central	56 (5.2)	5 (3.7)	
East	40 (3.7)	4 (3.0)	
North	122 (11.4)	12 (8.9)	
South	246 (22.9)	35 (25.9)	
West	141 (13.1)	22 (16.3)	

*Almaty city and Astana city were analysed as separate administrative units and were not included within the surrounding geographic macro-regions.

### National incidence trends of AA (2014–2024)

**Figure 1:**
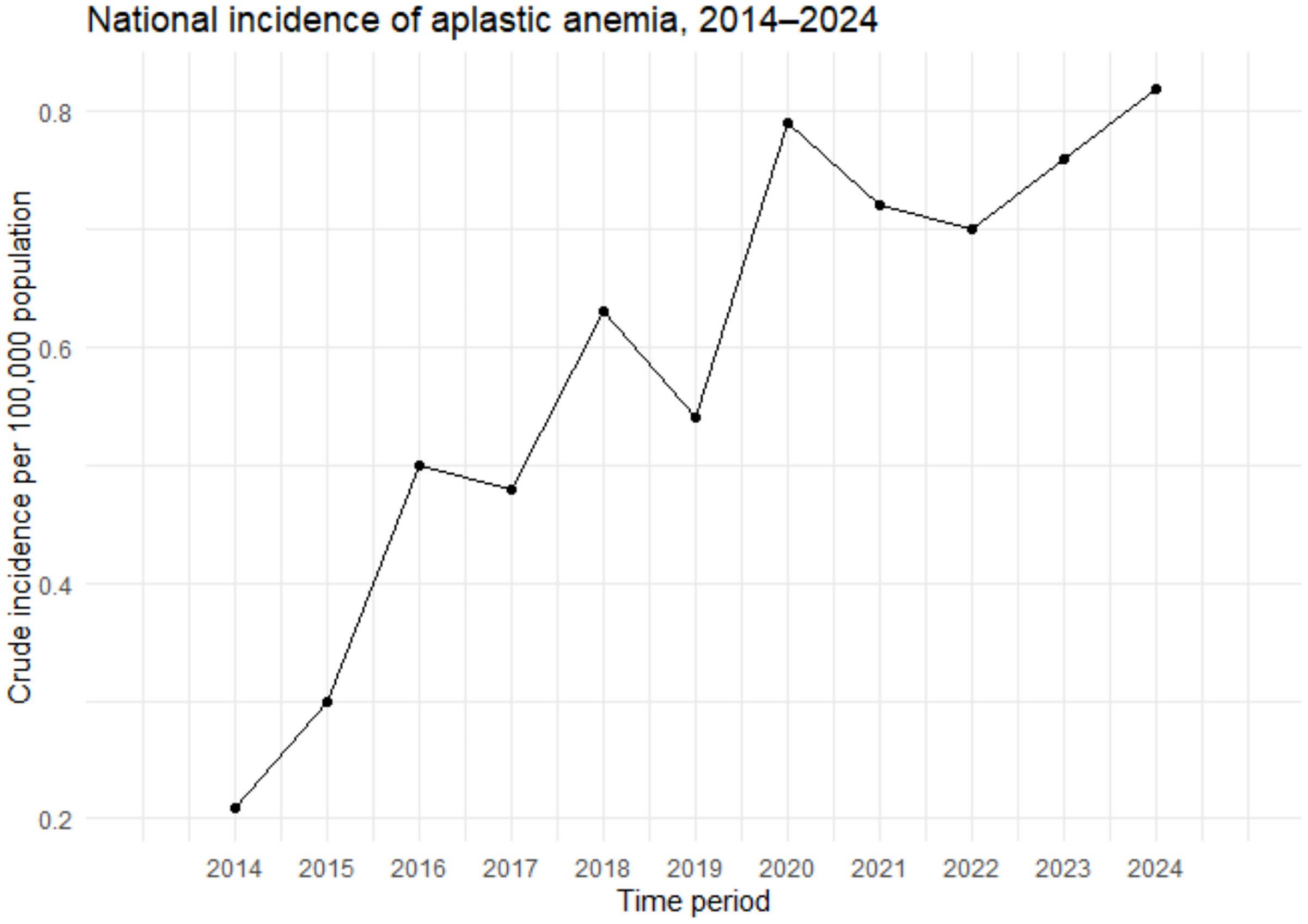
Temporal trends in the national incidence of aplastic anaemia, Kazakhstan, 2014–2024.

At the national level, the incidence of aplastic anaemia in Kazakhstan increased steadily over the 10-year period. Crude incidence rose from 0.21 per 100,000 population in 2014 to 0.50–0.53 per 100,000 between 2016 and 2019, driven by an increasing number of newly diagnosed cases (36 cases in 2014 to 98 cases in 2019) alongside moderate population growth. A notable rise occurred from 2020 onward, with incidence peaking at 0.79 per 100,000 in 2020 and continuing to increase to 0.82 per 100,000 in 2024, corresponding to 165 newly identified cases that year. Overall, these findings indicate a *gradual upward trend* in aplastic anaemia incidence over the past decade, reflecting both increased case detection and ongoing population changes (Figure 1).

**Figure 2:**
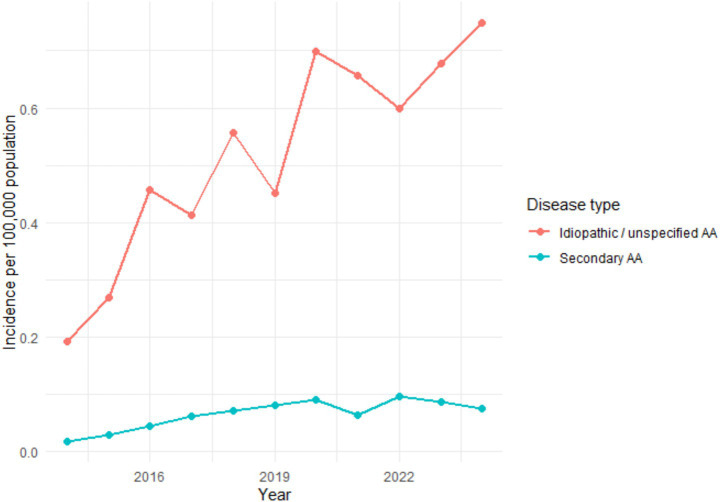
Temporal trends in the national incidence aplastic anaemia by diagnostic category, Kazakhstan, 2014–2024.

Further stratification by aplastic anaemia subtype demonstrated that the observed national increase was primarily driven by idiopathic or unspecified cases (Figure 2). The incidence of idiopathic/unspecified AA increased from 0.19 per 100,000 population in 2014 (33 cases) to 0.75 per 100,000 in 2024 (150 cases), with a marked rise beginning in 2016 and peaking at 0.70 per 100,000 in 2020. Although minor fluctuations were observed thereafter, incidence remained consistently higher than pre-2016 levels throughout 2020–2024. In contrast, secondary AA remained comparatively uncommon over the study period. Incidence increased modestly from 0.02 per 100,000 in 2014 (3 cases) to a maximum of 0.10 per 100,000 in 2022 (19 cases), with annual rates generally ranging between 0.06 and 0.09 per 100,000 in recent years. Overall, idiopathic/unspecified AA accounted for the vast majority of newly diagnosed cases each year and largely explains the upward trend in national aplastic anaemia incidence observed over the decade.

**Figure 3:**
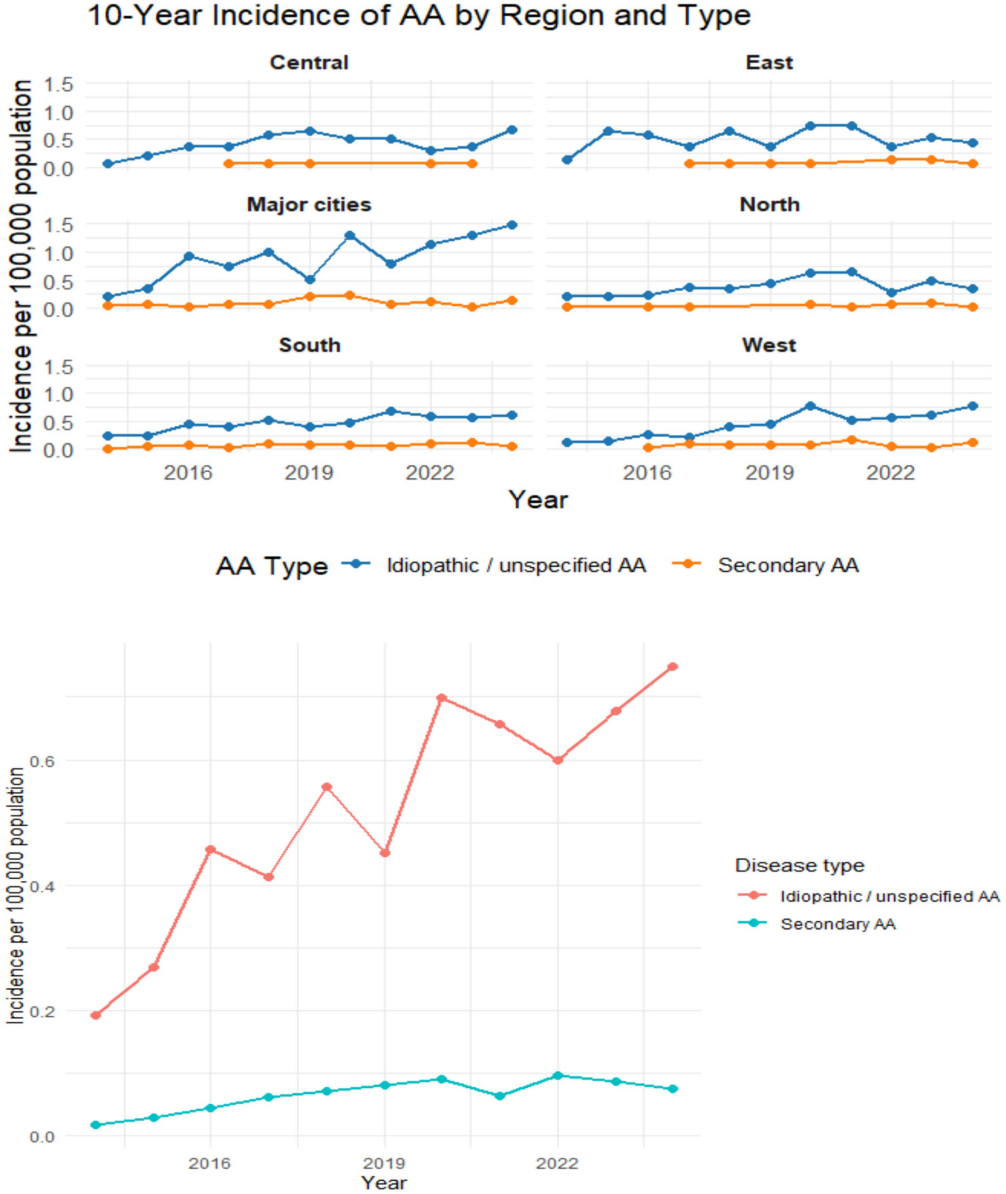
Regional Trends in the incidence of aplastic anaemia by region, Kazakhstan, 2014–2024.

Over the 10-year period, the incidence of AA varied notably across regions (Figure 3). Idiopathic or unspecified AA consistently exhibited higher incidence rates than Secondary AA in all regions. Major cities showed the highest incidence, peaking at 1.48 per 100,000 in 2024, while other regions ranged from 0.3 to 0.9 per 100,000. Secondary AA remained relatively rare, with modest fluctuations between 0.03 and 0.22 per 100,000. Temporal trends suggest a gradual increase in Idiopathic AA incidence across all regions, with the steepest rise observed in urban centers, whereas Secondary AA showed minimal changes over time. These patterns indicate that both AA type and geographic context contribute to the observed regional differences, potentially reflecting variations in population density, environmental exposures, healthcare access, or diagnostic practices.

## Discussion

This population-based longitudinal study provides comprehensive national evidence on the epidemiology of aplastic anaemia in Kazakhstan over the period 2014–2024. The findings demonstrate a clear and sustained increase in AA incidence over the past decade, largely driven by idiopathic or unspecified cases. Although AA remains a relatively rare hematological disorder, the observed upward trend suggests either a true increase in disease occurrence, improved case ascertainment and diagnostic capacity, or a combination of both factors. In addition, notable regional variation was observed, with the highest incidence rates reported in Almaty and Astana cities, potentially reflecting differences in healthcare accessibility, diagnostic infrastructure, referral patterns, and population density across regions.

Globally, AA is considered a rare disease, with reported incidence ranging from approximately 1.5 to 7 cases per million population per year, with higher rates observed in Asia compared to Europe and North America (4–12). The increasing incidence observed in Kazakhstan is therefore consistent with patterns reported in Asian populations, where higher rates have been attributed to environmental, genetic, and diagnostic factors (3). To our knowledge, this is the first national study describing long-term trends in the incidence of AA in Kazakhstan.

The predominance of idiopathic or unspecified AA (88.9%) in this study aligns with global evidence indicating that the majority of AA cases lack an identifiable cause, with idiopathic cases accounting for approximately 70–80% worldwide (3, 20). This highlights the ongoing challenges in identifying etiological factors and suggests that immune-mediated mechanisms may play a central role in disease pathogenesis.

The age distribution observed in Kazakhstan, with a relatively young median age, is also consistent with international studies reporting a bimodal age pattern, with peaks in adolescents/young adults and older individuals (12, 21). However, the slightly higher proportion of females in this study contrasts with global data suggesting roughly equal sex distribution, indicating potential differences in healthcare utilization or population structure (1, 14, 22). Young patients with AA face significant social, financial, and psychological challenges in addition to medical issues. Effective care requires clear communication, long-term follow-up, and strong support systems, including mental health and social support (21, 23). In Kazakhstan, PHC also plays an important role in health education for the populations they serve. Therefore, upon diagnosis, in addition to management by a general practitioner, primary health care should emphasize patient and family education through preventive departments. This approach has the potential to improve awareness, treatment adherence, and clinical outcomes.

Regional variation in incidence observed in this study mirrors global disparities, where differences in healthcare access, environmental exposures, and socioeconomic conditions influence disease burden. The higher incidence in urban centers in Kazakhstan likely reflects improved diagnostic capacity and reporting systems rather than true differences in disease occurrence, a phenomenon also described in international epidemiological studies. This discrepancy suggests that observed urban predominance may partly reflect detection bias rather than true epidemiological differences.

Considering that the risk of AA may be higher or underdiagnosed in rural areas, it is important for the healthcare system to strengthen services in these regions (14, 24). This is particularly relevant given the potential for underdiagnosis and delayed presentation in rural populations. In addition, targeted training of healthcare professionals in rural settings is needed to improve early detection, ensure stepwise and continuous care throughout the patient’s life course, and facilitate effective communication and information transfer within the healthcare system. Such measures are especially important, as timely diagnosis has been shown to significantly improve survival outcomes in patients with AA (25).

Overall, the upward trend in AA incidence in Kazakhstan is consistent with global observations suggesting that improved surveillance, diagnostic advancements, and increased awareness contribute to rising reported incidence rates. These findings highlight the importance of strengthening national surveillance systems and ensuring equitable access to haematology services. They also underscore the need for integrated public health strategies that combine enhanced surveillance, equitable access to care, and targeted education programs.

### Strengths and limitations

This study has several notable strengths. First, it is based on a large, nationwide dataset covering all administrative regions of Kazakhstan over a decade, providing a comprehensive and representative assessment of AA incidence. Second, the use of standardized ICD-10 diagnostic codes enhances the consistency and comparability of case identification. Third, the longitudinal design allows for the analysis of temporal trends and regional variation, offering valuable insights into evolving epidemiological patterns. Finally, the distinction between idiopathic/unspecified and secondary AA improves interpretability and contributes to a more nuanced understanding of disease aetiology.

Several limitations should be considered when interpreting the findings. First, the reliance on administrative and electronic medical record data introduces the potential for misclassification and diagnostic inaccuracies, particularly in distinguishing idiopathic from secondary AA. In addition, the database did not include information on bone marrow biopsy confirmation or centralized hematopathology review, limiting the ability to clinically validate AA diagnoses and assess diagnostic accuracy across regions. Given that the diagnosis of aplastic anaemia typically requires specialized hematological evaluation, including bone marrow examination, regional disparities in access to tertiary hematology care and expert pathology services may have influenced case ascertainment and reporting practices. As a result, the observed geographic variation in incidence rates should be interpreted with caution, as some differences may reflect variability in diagnostic infrastructure and healthcare access rather than true underlying differences in disease occurrence. Second, incomplete clinical information limited the ability to assess underlying causes, risk factors, and disease severity. Third, regional differences in healthcare access and diagnostic capacity may have resulted in underdiagnosis or underreporting in certain areas, potentially biasing incidence estimates.

Additionally, improvements in diagnostic practices and reporting over time may partly explain the observed increase in incidence, rather than reflecting a true rise in disease occurrence. The lack of data on environmental exposures, socioeconomic status, and treatment outcomes further limits the ability to fully interpret regional variations and causal pathways. Finally, as this study focuses on incidence, it does not capture disease prevalence, survival, or long-term outcomes, which are important for understanding the full burden of AA.

## Conclusion

This study demonstrates a clear and sustained increase in the incidence of AA in Kazakhstan between 2014 and 2024, predominantly driven by idiopathic or unspecified cases. The findings highlight notable regional variation, with higher incidence observed in urban centers, likely reflecting differences in diagnostic capacity and healthcare access. Overall, these results underscore the need to strengthen national surveillance systems, improve diagnostic accuracy, and ensure equitable access to haematology services across regions. Enhanced epidemiological monitoring and targeted public health strategies are essential to better understand disease patterns, address potential risk factors, and improve outcomes for patients with AA in Kazakhstan.

## Data Availability

The original contributions presented in the study are included in the article/supplementary material, further inquiries can be directed to the corresponding author.
